# Hydraulic stress parameters of a cased caddis larva (*Drusus biguttatus*) using spatio-temporally filtered velocity measurements

**DOI:** 10.1007/s10750-020-04349-0

**Published:** 2020-07-10

**Authors:** Johann Waringer, Simon Vitecek, Jan Martini, Carina Zittra, Stephan Handschuh, Ariane Vieira, Hendrik C. Kuhlmann

**Affiliations:** Division Limnology, Department of Functional and Evolutionary Ecology, University of Vienna, Althanstrasse 14, 1090 Vienna, Austria; WasserCluster Lunz, Dr. Carl Kupelwieser Promenade 5, 3293 Lunz am See, Austria; Institute of Hydrobiology and Aquatic Ecosystem Management, University of Natural Resources and Life Sciences Vienna, Gregor-Mendel-Straße 33, 1180 Vienna, Austria; Division Limnology, Department of Functional and Evolutionary Ecology, University of Vienna, Althanstrasse 14, 1090 Vienna, Austria; Institute of Hydrobiology and Aquatic Ecosystem Management, University of Natural Resources and Life Sciences Vienna, Gregor-Mendel-Straße 33, 1180 Vienna, Austria; Division Limnology, Department of Functional and Evolutionary Ecology, University of Vienna, Althanstrasse 14, 1090 Vienna, Austria; VetCore Facility for Research. Imaging Unit, University of Veterinary Medicine, Veterinärplatz 1, 1210 Vienna, Austria; Institute of Fluid Mechanics and Heat Transfer, TU Wien, Tower BA/E322, Getreidemarkt 9, 1060 Vienna, Austria

**Keywords:** Hydraulic stress parameters, Flow properties, *Drusus biguttatus*, Trichoptera larvae

## Abstract

By studying hydraulic stress parameters of larvae of the cased caddisfly *Drusus biguttatus* (Pictet, 1834) in a tributary of the Schwarze Sulm (Carinthia, Austria), we aimed on (1) detecting the flow properties of the spatio-temporally filtered velocity measurements taken, and (2) on defining the hydraulic niche of this caddisfly larva. For this, we took 31 measurement series lasting 30 to 300 s, yielding 2176 single velocity measurements. The probability density functions of the 31 data series were Gaussian or sub-Gaussian, and the mean recurrent interval between velocity maxima within a data series was only 15.00 s. As a consequence, the Trichoptera larvae studied have to face strong flow accelerations in short intervals which is a much higher stress than conventional mean velocity measurements would suggest. The hydraulic niche of *Drusus biguttatus* is defined by instantaneous flow velocities ranging from 0.04 to 0.69 m s^−1^, by drag forces from 13 × 10^−6^ to 3737 × 10^−6^ N, by Froude numbers from 0.13 to 1.20, and mostly by Reynolds numbers > 2000. Under such conditions, only 5.1% of the drag force is compensated by submerged weight, whereas the remainder has to be counterbalanced by the active efforts of the larvae to remain attached to the substrate.

## Introduction

Linking organismic responses of aquatic biota to their physical environment is the main research focus of ecohydraulics and hydraulic stream ecology (Ditsche-Kuru et al., 2011, 2012; Ditsche et al., 2013, 2014a, b; Maddock et al., 2013; Statzner et al., 1988; Steuer et al., 2009; Tay et al., 2015). On the applied side, the methodological concepts used in ecohydraulics range from drag force reduction in high-speed swimmers (Sadeghizadeh et al., 2017) to improvements in submarine design (Dawson, 2014). For macroinvertebrates, stream hydraulics are seen as a major determinant of zonation patterns (Statzner & Higler, 1986), and stream ecology and hydraulic engineering, along with geomorphology, should be seen as key elements of an integrated, interdisciplinary river science (Rice et al., 2009).

One striking observation in the study of interactions between stream macroinvertebrates and their surrounding medium is the paradox that most body shapes of aquatic biota seem to be not well adapted to hydraulic stress (Statzner et al., 1988; Waringer, 1989a, b, 1993) although those taxa have to cope with considerable hydrodynamic forces (Statzner & Holm, 1982; Ditsche-Kuru et al., 2010). This ecological paradox also fully applies to the Drusinae, a caddisfly subfamily restricted to running waters covering the Eurasian mountain ranges from the Iberian Peninsula to the Iranian Highlands. Driven by limited food availability that is highly variable in space and time, the need of predator protection and the ever-changing hydraulic stress of their lotic environment, species have evolved an amazing diversity of body morphologies. Meeting such a multitude of evolutionary constrains often provokes ecological trade-offs. For example, the flocculent hair cover in filtering Drusinae attracts prey by mimicking natural substrate (Bohle, 1983), and the sophisticated pronotal structures enable blocking the case against predators when larvae withdraw (Waringer et al., 2015). Those ecological benefits, however, are offset by heavily changing flow fields around larvae, thereby increasing hydraulic stress (e.g., Vitecek et al., 2015; Waringer et al., 2007a, b, 2010, 2013a, b; Waringer & Graf, 2011). Such examples tell us that in many cases morphological structures must not be interpreted as flow adaptations, but as ecological trade-offs triggered by a multitude of other evolutional needs. This also fully applies to *Drusus biguttatus* (Pictet, 1834), a member of the Drusinae group of epilithic grazers, which lack terminal teeth on their mandibles. From a fluiddynamical perspective, *Drusus biguttatus* larvae in their typical stream habitats can be seen as near-wall, submerged cylinder-like bodies exposed to a moving fluid with their longitudinal axis aligned with the mean flow direction and their heads facing towards the flow. Based on the average body height of the larvae when clinging with their legs on the bottom of the stream which is up to 7 mm above the substrate, Drusinae larvae stay always well outside of the viscous sublayer and reach well into the log-law turbulent layer. This turbulent flow causes random forces on the larvae characterized by a mean stress and a fluctuating part. The latter is composed of rapid acceleration and retardation processes with severe ecological implications: hydraulic stress can be seen as a continuous series of hydraulic strokes on the projected surface of benthic macroinvertebrates which may take much more effect than a theoretical continuous impact without any fluctuations in magnitude, as suggested by commonly used descriptors of hydraulic stress based on mean flow velocity.

In the present study, we aim to provide new insights into this paradigm using spatio-temporally filtered velocity measurements. Specifically, (1) we wanted to elucidate the magnitude of turbulence intensity representing the anisotropic macroturbulences created by small-scale irregularities of the stream bed, and (2) to relate this information with stress parameters describing the hydraulic niche of a Drusinae caddisfly larva.

## Materials and methods

### Field measurements

The field measurements were made in a small, springfed, 50-m long, first-order tributary of the Schwarze Sulm on Koralpe, Weinebene, near Gösler Hütte at 1580 m above sea level (Carinthia, Austria). The steep (slope = 0.1006 m m^−1^), shallow, clean, fast flowing, summer-cold mountain brook was 50 to 100 cm wide and bordered by meadows (water temperature = 10.5°C). Measurements were conducted at the sites of fifth instar larvae of *Drusus biguttatus*. This limnephilid caddisfly is distributed over the Alps, the Balkans, the Carpathians, and the Central and Western European Highlands (Neu et al., 2018). In final instars of *Drusus biguttatus* larvae, head widths range from 1.45 to 1.65 mm (Waringer & Graf, 2011). Their cases are slightly curved, slightly conical, with mean anterior case diameters (± 95% confidence limits CL) of 3.16 ± 0.27 mm (range = 2.8–4.0 mm), creating mean projected frontal areas (± 95% CL) of 7.92 ± 1.28 mm^2^. Cases consist of mineral particles (sand grains of mixed size; mean case length ± 95% CL = 10.67 ± 1.28 mm). In their typical habitat, the longitudinal body axis is generally aligned with flow; while the abdomen of the larva is inside of its case. Head and thorax protrude from the anterior case opening, adding 40% of case length to the longitudinal axis of the larva. Depending on body posture, the dorsal case outlines of the larvae were 6 to 7 mm above the sediment surface. We chose larvae located at the top center of horizontal flat mineralic sediment particles with grain sizes > 20 cm (megalithal) and water depth of 10 mm.

**Fig. 1:**
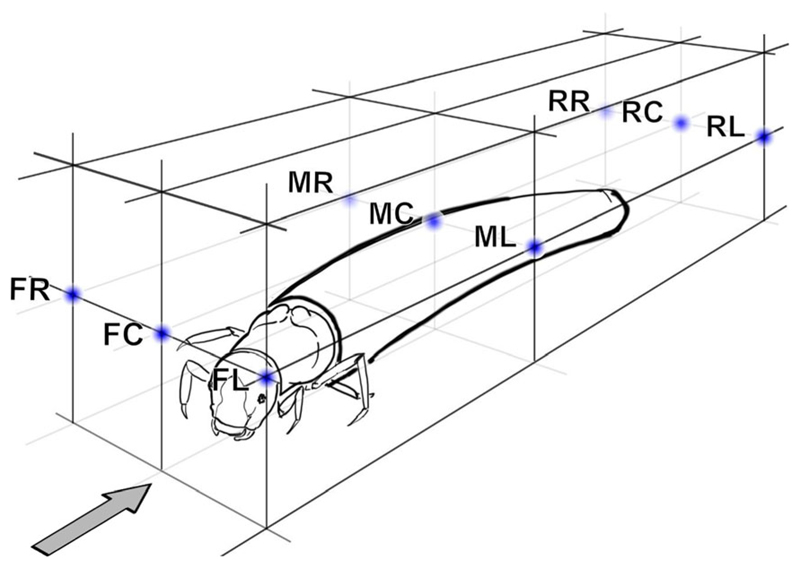
Setup for spatio-temporally filtered flow velocity measurements at the site of a *Drusus* larva. Acronyms define the positions of the center of the Schiltknecht MiniWater 20 Micro propeller meter used (propeller diameter = 10 mm). Distance between monitoring points FC – FL = 10 mm; distance between front, middle and rear monitoring points vary in accordance with larval length. *F* front, *M* middle, *R* rear, *R* right, *C* center, *L* left, *arrow* flow direction

Flow velocity was measured to the nearest 0.01 m s^−1^ with a Schiltknecht MiniWater 20 Micro propeller meter (propeller diameter = 10 mm; time resolution = 1 measurement per s; measuring accuracy = ± 3.5%) held firmly in place by a custom-built tripod support. The diameter of the measuring head was only slightly smaller than the water depth. It was positioned such that the frame of the casing nearly touched the bottom. This way the measured velocities can be considered good approximations to the vertically averaged velocity. Measurements were either single-point measurements at a larval site with the center of the propeller at position FC [front center] (Fig. 1), or series of measurements with up to 8 positions around the same larva (positions FR [front right], FC [front center], FL [front left], MR [mid right], ML [mid left], RR [rear right], RC [rear center], RL [rear left]; Fig. 1). Each measurement consisted of a time series with sampling rate of 1 per second, lasting 30 to 300 s recorded by a data logger. In total, 31 time series yielding 2176 single velocity measurements were taken.

### Biometric parameters

The maximum head width, body length, case length, and maximum (anterior) case diameter of 11 final instar larvae (head width ≥ 1.36 mm) of *Drusus biguttatus* were measured in the laboratory under a binocular microscope to the nearest 0.01 mm. Staging of the larvae was made by comparing maximum head width with data given by Waringer & Graf (2011). Since head and pronotum of larvae fit tightly into the anterior case opening, the calculation of the (projected) frontal surface area of each larva was based on anterior case diameter. An approximation to total fresh weight (larva + case; to the nearest mg) was obtained after removing excess conservative fluid (70% Ethanol) with filter paper. In order to measure the total volume, larvae (in cases) were dropped into a burette partly filled with water; the difference in water level (to the nearest 0.01 ml) was equal to the total volume.

Adhesive friction *F*_a_ is typically expressed in terms of the weight of the submerged body and is given by

(1) $$F_{\text{a}}=f\;Vg\left(\rho_{1}-\rho \right),$$

where *V* is the volume of the larva plus case (m^3^), *g* is the acceleration due to gravity (9.81 m s^−2^), *ρ*_l_ is the density of the larva + case (kg m^−3^), and *ρ* is the density of water (kg m^−3^). The non-dimensional friction factor *f* for cases constructed of mineral particles on a mineral substrate is 0.69 (Waringer, 1989, 1993). Biometric data are summarized in Table 2.

### Hydraulic stress parameters

In the present study, liquid depth was approximately in the range of the height of the larva; therefore, the same mean flow velocity data could be used for mean velocity in the liquid layer and the mean velocity the larva is facing, as well as for all velocity-derived stress parameters.

The drag force *F*_d_ (N) exerted by the flow is given by

(2) $$F_{\text{d}}=C_{\text{d}}A\rho u^{2}/2,$$

where *A* is the projected frontal area of the larva, based on the mean anterior case diameter without taking into account the differences in the larval head shapes (m^2^), *ρ* (kg m^−3^) is the density of water which is temperature-dependent (*ρ*_10.5°C_ = 999.681 kg m^−3^), *u* is the mean flow velocity (m s^−1^) over area *A*, and *C*_d_ is the drag coefficient which is dimensionless (Waringer, 1989); for fifth instar larvae of genus *Drusus* with their longitudinal axis aligned with the flow direction, *C*_d_= 2 (experimental data given by Waringer 1993).

The Froude number *Fr* quantifies the relationship between the mean kinetic energy (*u*, as mean velocity) and the potential energy gain across the water depth *d*. *Fr* is used as another key descriptor of hydraulic stress acting at macroinvertebrates on the streambed and given by

(3) $$Fr=u/{\left(dg\right)}^{0.5},$$

where the term (*dg*)^0.5^ effectively is the phase velocity of gravity waves in shallow water. *Fr* is dimensionless, with subcritical flow indicated by *Fr* < 1.0, critical flow by *Fr* = 1.0, and supercritical flow by *Fr* > 1.0 (Statzner et al., 1988). In a well-known analogy, ‘supercritical’ is the equivalent to ‘supersonic’ with *Fr* taking the role of the Mach number in unidirectional flow.

Finally, the dimensionless organismic Reynolds number *R* is given by the equation (Vogel, 1981; Waringer, 1993):

(4) R=ul/v,

where *v* is the temperature-dependent viscosity of water (*v*_10.5°C_=1.31 × 10^−6^ m^2^ s^−1^), *u* is the mean flow velocity (m s^−1^), and *l* (m) is the length of the larva when their longitudinal axis is aligned with flow which was always the case in our study.

## Results

### Properties of spatio-temporally filtered velocity

**Fig. 2:**
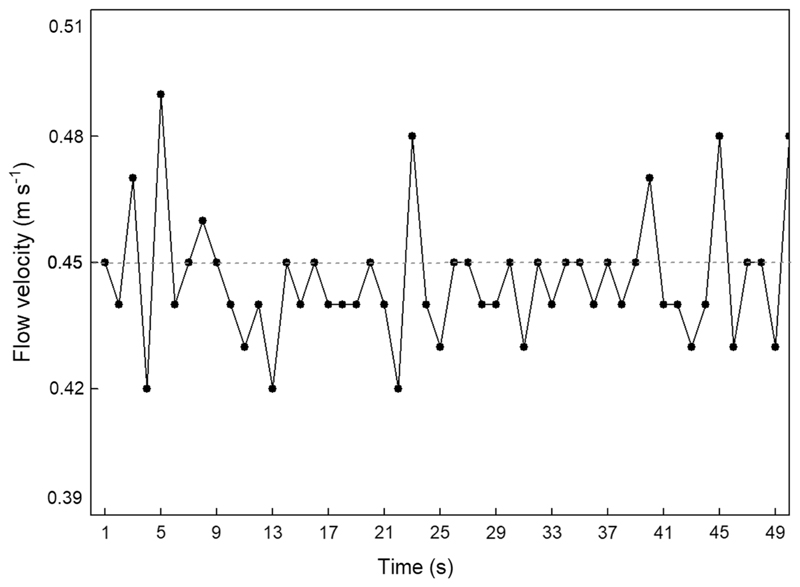
Detail of spatio-temporally filtered velocity measurements (velocity resolution = 0.01 m s^−1^) over a period of 50 s (time resolution = 1 measurement per s) for larva #7. Mean velocity is indicated by a dashed line

The measured velocity data are time-dependent and capture the dynamics of the slow time scales due to the temporal filtering provided by the sampling with 1 Hz. When plotting flow velocity versus time (Fig. 2), the most abundant patterns observed in the 31 recorded data series consisted of pulsed, sharp velocity changes at the resolution limit of 1 s. Less frequently, velocity peaks persisted up to 5 s over measurement time periods (Fig. 2). Mean velocities averaged over the whole fluid layer, together with root mean square values and absolute maxima and minima for the 31 data series are given in Table 1. The data obtained on different measuring positions around the same larva varied, without a systematic dependence on the position of the probe. This reflects the huge influence of small local roughness elements on the streambed.

**Fig. 3:**
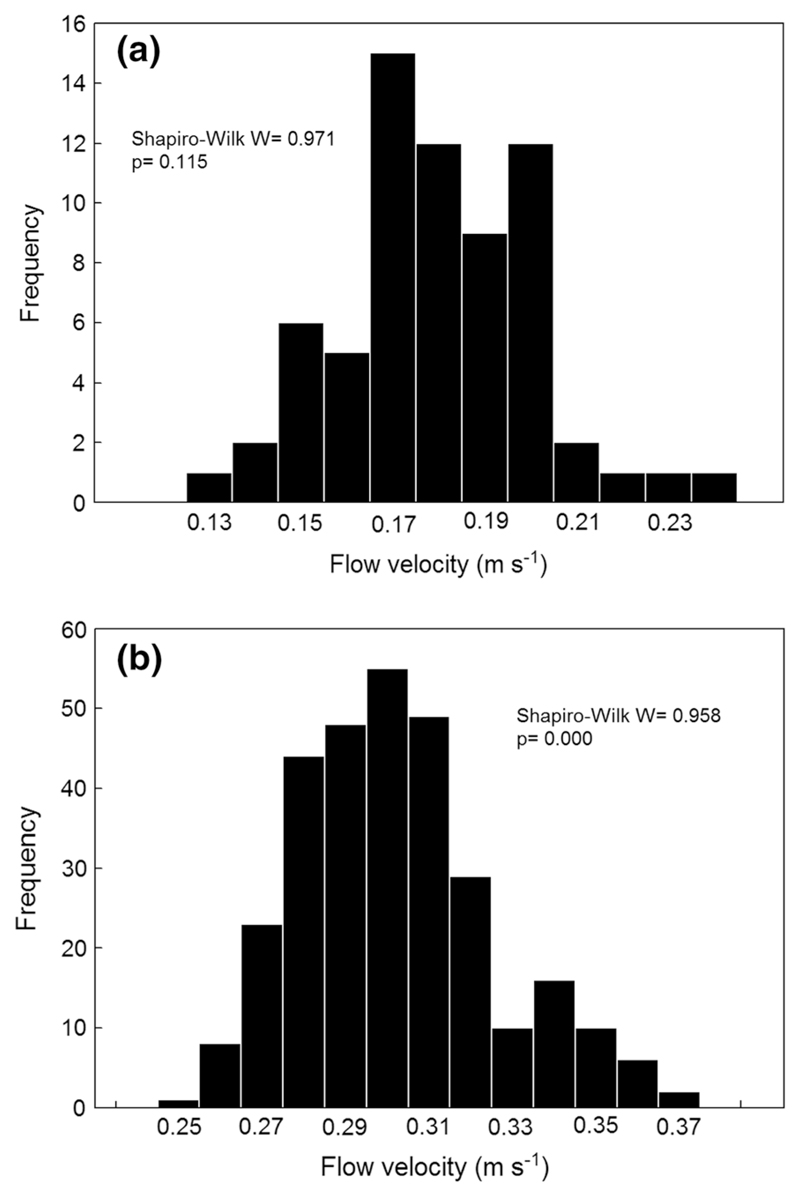
Frequency distributions of two series of spatio-temporally filtered flow velocity data (1 measurement per s) a over 67 s at the site of larva #6 (probe position: front left) and b over 301 s at the site of larva #8 (probe position: front center). Shapiro–Wilk’s W test of normality is not significant for normal distribution (a), but significant for (b) (*P* < 0.05) where the hypothesis that the distribution is normal is rejected

Two examples of frequency plots of single flow velocity measurements for series of 67 and 300 s duration are shown in Fig. 3. Shapiro–Wilks W tests of normality were used for analyzing distribution patterns of the 31 recorded velocity data series. In ten series, the W statistic was not significant (*P* > 0.05). Therefore, the hypothesis that the distribution is normal was confirmed (e.g., Fig. 3a); however, this hypothesis was rejected in the remaining 21 series, reflecting the non-homogeneous and anisotropic conditions within the large scales of the flow fields measured (e.g., Fig. 3b).

**Fig. 4:**
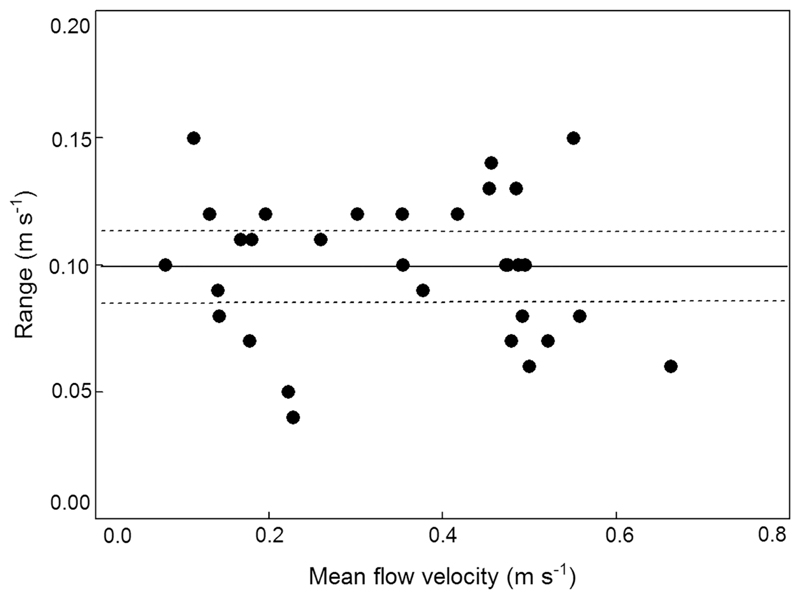
Relationship between mean flow velocity (m s^−1^) and range of 31 spatio-temporally filtered flow velocity series of 30 to 301 s duration (intervals = 1 s); showing mean (black line) with 95% CL (dotted lines)

**Fig. 5:**
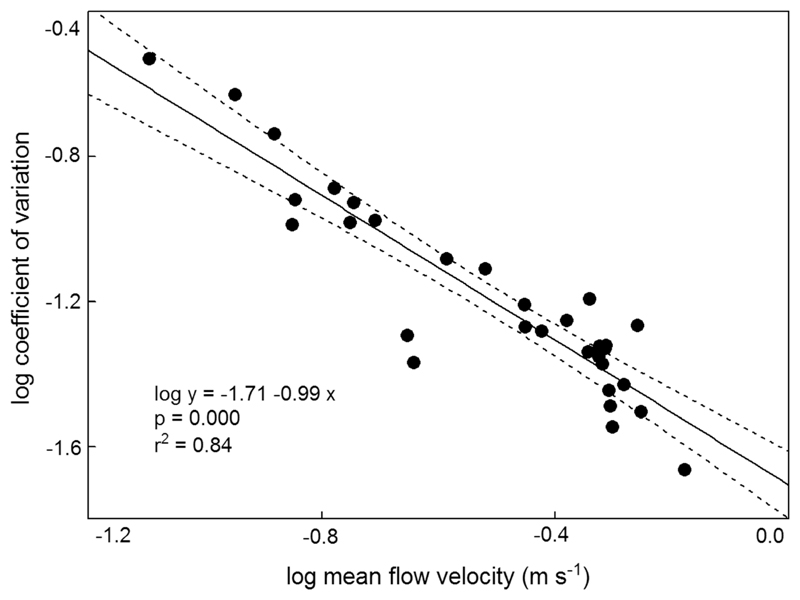
Relationship between mean flow velocity (log m s^−1^) and coefficient of variation (log) for spatio-temporally filtered velocity data (intervals = 1 s; 31 measuring runs); showing regression line and 95% CL confidence bands

Velocity ranges (= range between absolute maxima and minima per series) for the 31 data series of 30 to 300 s duration are shown in Fig. 4. There was no correlation between range and duration of a given velocity series, nor was there any correlation between mean flow velocity and range which varied from 0.04 to 0.15 m s^−1^ (arithmetic mean (± 95% CL) = 0.10 ± 0.01 m s^−1^; Fig. 4). The same applied to standard deviations which varied from 0.010 to 0.030 m s^−1^ (mean (± 95% CL) = 0.020 ± 0.002 m s^−1^). In terms of percentage, the standard deviation (mean (+ 95% CL) was 7.90 + 2.96% of the mean for the 31 data series. In order to explore the extent of variability of standard deviations in relation to the mean, we calculated the coefficient of variation. Due to the fact that standard deviation is divided by the mean, a plot of means versus coefficients of variation shows a decreasing linear relationship on a log–log scale when standard deviations remain fairly constant (Fig. 5).

**Fig. 6:**
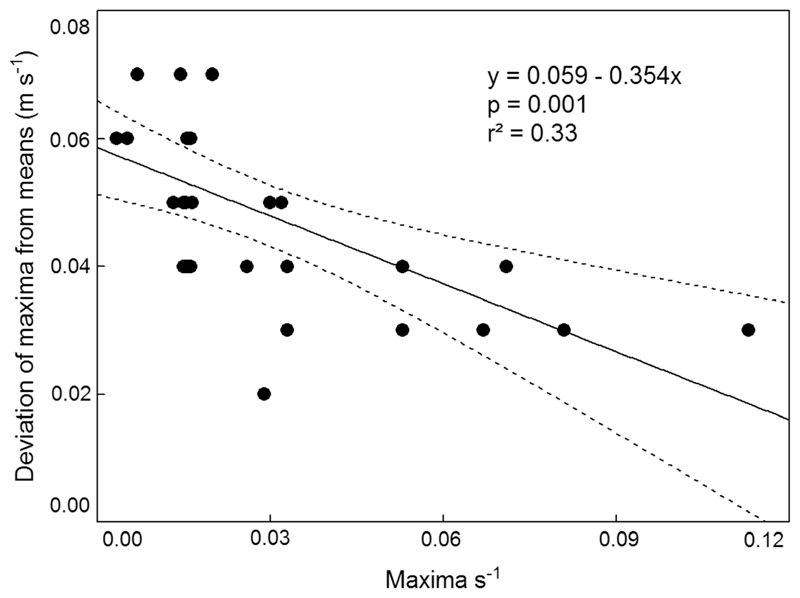
Relationship between the frequency of flow velocity maxima s^−1^ for each spatio-temporally filtered data series and the deviation of flow velocity maxima from mean flow velocity of the respective data series; showing linear regression line and 95% CL confidence bands

To characterize the periodicity of the strongest stress peaks on the larva, we define the “recurrent interval” as the mean time interval between two successive major maxima of the velocity measured within the total measurement period. A major maximum is a local maximum which deviates less than ± 0.01 m s^−1^ from the global velocity maximum in the total measurement interval. The recurrent interval within each of the 31 data series is an upper bound of hydraulic stress impacts faced by the larvae and ranged from 1 to 45 s (mean ± 95% CL = 15.00 ± 5.26 s). Flow maxima (mean ± 95% CL) were 0.04 ± 0.01 m s^−1^ higher than the respective mean velocities (range 0.03–0.07 m s^−1^). The duration of velocity measurements (resolution Δ *u* = ± 0.01 ms^−1^) had no significant effect on the recurrent intervals of absolute maxima (Mann–Whitney U-test; *P* > 0.05). However, there was a significant (*P* = 0.001) negative relationship between the frequency of velocity maxima per second and the deviation of maxima from mean velocities of the respective time series: the higher the frequency, the lower the maxima (Fig. 6).

### Hydraulic stress parameters

Extremal flow velocities (at any instant of time given the temporal resolution of 1 s) at the sites of *Drusus biguttatus* larvae ranged from 0.04 (larva # 4, propeller meter head position middle left, and larva # 6, rear left) to 0.69 m s^−1^ (larva # 4, rear left); the mean velocity for all 9 larvae and all measuring positions (pooled data) was 0.35 ± 0.01 m s^−1^ (Table 3). In addition to the standard measurement head position at front center, additional flow velocity measurements were made at up to 8 positions around three larvae (Fig. 1). Generally, 95% confidence limits were very narrow when pooling all measuring positions around those larvae (0.16 ± 0.00 m s^−1^ in larva #6, 0.45 ± 0.01 m s^−1^ in larva #9, 0.45 ± 0.02 m s^−1^ in larva #4; Table 3). This indicates that one series of spatio-temporally filtered velocity measurements at front center is an adequate effort for field studies.

The extreme values of drag exerted on the larvae of *Drusus biguttatus* varied between 13 × 10^−6^ N and 3737 × 10^−6^ N. In terms of means per data series, the mean drag varied by a factor of 10 for the nine larvae, ranging from 197 × 10^−6^ to 1798 × 10^−6^ N. Mean drag for pooled data including all 31 measuring runs (± 95% CL) was (1120 ± 34) 9 10^−6^ N (Table 3). The force resisting this drag is adhesive friction due to the submerged weight of the larva (mean = 8.46 ± 5.43 mg), which was equivalent to (57.27 ± 36.74) 9 10^−6^ N (Table 2). This illustrates the fact that on the streambed the larvae have to actively withstand almost the whole drag force. In 3 out of 31 data series, adhesive friction was higher than drag, indicating that submerged weight (modified by the friction factor *f*) alone was able to fully stabilize the larva in its hydraulic environment, without the need to spend any active effort to remain stationary on the stream bed. This was the case in larva #4 (position ML); at this larval position, the mean of the data series was up to (55.08 ± 9.23) 9 10^−6^ N, and 63% of the data points were well below the threshold of adhesive friction. In larva #6 (positions RR and RL), the means of 139.96 ± 11.44 and 105.34 ± 11.65 ( × 10^−6^ N), respectively, were significantly higher than adhesive friction, with only a small fraction (≤ 14%) of data points below this threshold.

**Fig. 7:**
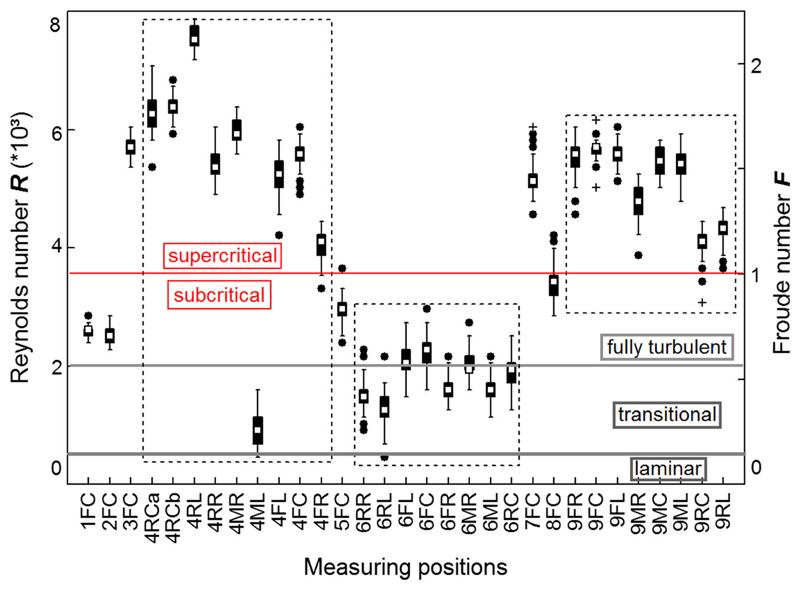
Box plots of Reynolds and Froude numbers (*R*, *Fr*) acting on fifth instar larvae (numbered 1 to 9) of *Drusus biguttatus* in the field with their longitudinal axis aligned with flow; showing the measuring positions of the propeller meter head (codes as in Fig. 1), and *R* and *Fr*, respectively (dimensionless). In addition to the measuring position at front center (FC), additional data were taken around larvae # 4, 6, and 9 (boxes with dotted lines). Gray lines divide *R* values in the laminar, transitional, and fully turbulent flow range, a red line divides *Fr* values in the sub- and supercritical range. Data for measuring positions 4FR, 5FC, and 8FCB indicate a transition of sub- to supercritical Froude numbers. White rectangles = means, black bars = 25/75% quartiles, whiskers = range without outliers, black dots = outliers, black crosses = extremes

Mean Froude numbers (± 95% CL) per larva varied from 0.50 ± 0.01 in larva #6 to 1.60 ± 0.01 in larva #3; as *d* was constant (= 10 mm) in all our measurements, this indicates that hydraulic effects of the sediment structures around the larvae play an important role on Froude numbers via the mean flow velocity. For pooled data of the 31 measuring runs, *Fr* was 1.11 ± 0.02. Highest *Fr* values were observed in larva #4 (measuring position RL: 2.20), lowest values of 0.13 in larvae #4 (ML) and 6 (RL). In 4 out of 9 larvae mean *Fr* values were well in the supercritical state > 1.0 (Fig. 7). In addition, 4 data series (4 FR, 5 FC, 8 FC, 9 RC) indicated a transition between sub- and supercritical (Fig. 7).

The flow in streams is always characterized by *R* > 1. Therefore, hydrodynamic inertia forces always play a role for the velocity field. Due to the complex bottom topography in a natural environment the flow is always chaotic, i.e., three-dimensional and time-dependent. As the Reynolds number increases, more and more spatial and temporal scales arise in the flow field and, according to Dingmann (1984), fluvial flows are considered fully turbulent for Reynolds numbers exceeding 2000 and transitional in the range of 500–2000. In our data, water depth (= 10 mm) was always close to the length of the larva in its case. Therefore, the organismic Reynolds number matches the Reynolds number of the incoming flow where the length parameter of Eq. (4) is water depth instead of larval length. We used the same mean velocity *u* obtained from our measurement for the definitions of the Froude and the Reynolds number. This is justified, because the height *h* of the larva is of the same order of magnitude as the depth of the liquid *d*. In a total of 81% of the time-dependent data series, organismic *R* was well in the fully turbulent range, with the remainder being transitional and none in the laminar range (Fig. 7). When comparing minima of organismic *R* of the 31 data series, only a single data point satisfied *R* < 500. In addition to hydraulic stress experienced by the larvae themselves, they generate additional turbulent fluctuations in their wake, thereby influencing the hydraulic field in their vicinity.

## Discussion

For remaining stationary on the stream bed and to prevent drift entry, benthic organisms have to resist hydraulic stress which is based both on mean flow, strongly modified by fluctuations of the velocity field. In order to get information on both components, measurements consisting of sampled data series lasting for extended periods of time are necessary. The best method for time-resolved velocity measurements would be to use hot wire velocimetry. However, such devices are rather fragile, and the probe may fail if it runs dry temporarily. In a remote high-alpine environment, we rely on a sturdier equipment where a small propeller meter seems to be a good compromise between sturdiness and resolution; such a device can easily expand this method also to higher water depths and more heterogeneous substrates. Due to the measuring device used, the measured flow velocity data represent a spatial and temporal filtering of the true velocity. Spatial filtering exists, because the Kolmogorov scale of the turbulences is thousand times smaller than the diameter of the propeller, over which we average the velocity. A temporal filtering exists, because the response time of the propeller (= 1 s) is larger than the shortest time scales in the turbulent flow: the time it takes for a small eddy to cross the turbine plane is equivalent to 1.6 × 10^−3^ s. We need to keep this filtering in mind when evaluating the measurements. Our data are time-dependent and capture the dynamics of the slow time scales. This is due to the temporal filtering (low pass filter) provided by the sampling with 1 Hz, reflecting the response time of the probe.

The flow velocity fluctuations observed in our spatio-temporally filtered velocity measurements (Fig. 2) reflect the fact that Drusinae larvae in their lotic environment reach well into the log-law layer in which turbulent fluctuations must be taken into account (Kuhlmann, 2014). This means final instar *Drusus biguttatus* larvae are not only exposed to the hydraulic stresses caused by the mean flow. Rather, the fluctuating part of the velocity field is very important, because the strong fluctuation not only cause fluctuating stresses, but also significant mean forces via the Reynolds stresses. Due to the limited time resolution of the propeller meter used (= 1 s), we are aware that the fluctuations observed in Fig. 2 represent anisotropic macroturbulence consisting of eddies created by small-scale irregularities of the stream bed (Matthes, 1947). Large-scale fluctuations affected by the geometry of the bottom topography and the anisotropy due to changing directions of the mean flow also shaped distribution patterns of velocity pulsations (Fig. 3).

Turbulence intensity *I* is generally considered as the standard deviation of a number of *n* instantaneous velocity fluctuations *u* (m s^−1^) relative to the mean downstream velocity *ū* (m s^−1^; Gordon et al., 1992; Hickin, 2004). In our data set (Table 1), standard deviations remained fairly constant over the range of mean flow velocities observed per data series, ranging from 0.010 to 0.030 m s^−1^. Since the mean coefficient of variation (± 95% CL) was 7.9 ± 2.3% for the 31 data series, these fluctuations are quite large. We found *I* to be < 5.0% (medium turbulence) in 14 and > 5.0% (high turbulence) in 17 out of 31 cases. This indicates a high proportion of high-speed flow within the complex geometries of mountain streams caused by the irregular bottom topography, which differs from standard smooth geometries used in most turbulence experiments.

This unsteadiness of flow caused by local acceleration and retardation processes poses severe ecological implications: turbulent flow causes a kind of random force on the larvae, characterized by a mean value (i.e., larvae must withstand a mean stress), and a fluctuating part. Given the resolution of the device used, larva #4, for example, had to face strong flow accelerations on average every 2.5 s that would provoke drift entry much more effectively than the associated conventional mean velocity measurement of 0.45 m s^−1^ would suggest. In this light it could be a good idea to re-think published drift entry data based on mean velocities; the same applies to other commonly used descriptors of hydraulic stress based on mean flow velocity, such as drag, Froude and Reynolds numbers.

When analyzing pooled data of all data series, *Drusus biguttatus* was able to withstand maximum instantaneous flow velocities up to 0.69 m s^−1^ without being dislodged from the substrate which is the highest specific velocity measured for this species we are aware of. In fact, this is much higher than published instantaneous threshold velocities for drift entry in other caddisflies, e.g., 0.28 ± 0.01 m s^−1^ in fifth instar and 0.13 ± 0.01 m s^−1^ in first instar larvae of *Allogamus auricollis* (Pictet, 1834) (Waringer, 1989). The highest threshold for drift entry for caddisflies so far has been reported for final instar larvae of *Silo nigricornis* (Pictet, 1834), which is up to 1.26 m s^−1^ (König & Waringer, 2008). Given the risks associated with drifting, such as mechanical injuries by smashing against boulders, increased fish predation, or the increased possibility of landing in unfavurable microhabitats (Waringer, 1992), withstanding the ever-changing flow stresses due to the velocity fluctuations is of vital importance for benthic invertebrates in streams. The reasons why benthic animals expose themselves to high flow velocities are manifold. According to the data available, one of the driving forces is feeding behavior which heavily depends on feeding type: *Drusus biguttatus* is a typical scraper (Vitecek et al., 2015) feeding on epilithic algae and biofilm, which, in turn, reach their highest densities midstream where hydraulic stress and water flow are highest (König & Waringer, 2008).

For pooled data including all 31 series of measurements of the present study, we observed a mean drag of (1120 ± 34) × 10^−6^ N. In order to prevent dislocation and drifting, benthic macroinvertebrates have to withstand this drag force either by evolving heavy bodies, cases or shells, large claws or effective leg muscles to attach to the substrate. It is also conceivable that *Drusus biguttatus* may use its silk to fasten the case to the substrate, but we did not explore this possibility. The effectiveness of such evolutionary achievements, however, is counteracted by the Archimedes’ principle where immersed bodies experience hydrostatic lift (buoyancy) which is equivalent to the density of the fluid times the volume of the immersed body (− *ρV*). This lift force reduces the submerged weight of last instar larvae of *Drusus biguttatus* by 82% (8.46 mg submerged weight versus 47.64 mg fresh weight; Table 2) and heavily impacts drag resistance in order to remain stationary on the stream bed and avoid drift entry. Under this perspective, the heavy cases constructed of mineral particles of mixed size significantly add to submerged weight, since the additional amount of frontal projected area is negligible when compared to the projected area of the larvae alone. On average, only 5.1% (range 3–29%; Tables 2, 3) of the drag force is compensated by submerged weight in *Drusus biguttatus*, whereas the remainder has to be counterbalanced by the active efforts of the larvae to remain attached to the substrate, such as muscular strength and the effectiveness of claw usage. In *Silo nigricornis*, where lateral ballast stones are integrated into the case design, this compensation is up to 40% (König & Waringer, 2008), which greatly expands the area of drift-save microhabitats: field studies have shown that in *Allogamus auricollis* and *Silo nigricornis*, 0% of the population was out of their range of adhesive friction on the stream bed. As a consequence, those species were significantly (*P* < 0.001) under-represented in the drift. On the other hand, 67% of the population of *Drusus biguttatus* was out of its range of adhesive friction, and this species was significantly (*P* < 0.001) over-represented in driftnet samples (Waringer, 1989b and Waringer, unpublished data).

Another force modifying adhesive friction is the hydrodynamic lift force as a consequence of the Bernoulli principle. Basically, the lift force is an inertial effect and arises due to circulation around an immersed body. In very small aquatic biota, viscous drag forces become more important than lift forces. In the typical size range of aquatic insect larvae, however, lift forces must not be neglected, and this effect becomes even more important when body shapes are dorsoventrally flattened: Weissenberger et al. (1991) showed that in flattened ephemeropterans, such as *Epeorus* sp., the lift force may be twice as high as pressure plus viscous drag. On the other hand, *Ecdyonurus* sp., another flattened mayfly species, is able to produce negative hydrodynamic lift forces by means of its large head shield which can be used to direct the flow in such a way as to press the body against the substrate. In benthic animals that are less or not at all dorsoventrally flattened, pressure drag by far outweighs lift force. For example, in the plecopteran *Perla bipunctata* Pictet 1833, drag was found to be 0.25 mN, but lift force was actually zero at a current speed of 40 cm s^−1^ (Weissenberger et al., 1991), a situation which is also applicable to *Drusus biguttatus*.

The drag coefficient *C*_d_ is defined as the ratio of the actual measured drag force to the reference force which results from the dynamic stagnation pressure acting on the representative cross-sectional area A of a body. For small submergence depths, *C*_d_ typically has a significant maximum for *Fr* < 1 and decreases strongly for larger *Fr*. This effect vanishes if the submergence distance becomes so large that surface waves become insignificant. What makes the drag force actually measured different from the reference force are effects of body shape, and the turbulent fluctuations in the typical habitats of *Drusus biguttatus*. Typically, *C*_d_-values may be lowered if the object has a long and tapering tail (Vogel, 1981). In *Drusus biguttatus* larvae, the rear diameter of cases is distinctly smaller than the front diameter, which can be considered as a kind of streamlined body shape. With their longitudinal axis aligned with flow, separation of the boundary layer can be prevented and the pressure on the downstream side of the body can increase more than in a separated flow. This results in a counteracting force, thereby reducing the drag. For fifth instar larvae of *Drusus* species (longitudinal axis aligned with flow), *C*_d_-values of 2.0 were obtained by experiments in artificial stream channels in the laboratory (Waringer, 1993). Weissenberger et al. (1991) reported *C*_d_-values of 0.9–1.0 for flattened ephemeropterans, 1.0 for the non-flattened ephemeropteran *Baetis* sp., and 1.9 for the plecopteran *Perla* sp.

In *Drusus biguttatus*, *Fr* values covered the whole range of subcritical, critical, and supercritical flow, with mean *Fr* (pooled data for all 31 series) well in the supercritical stage (1.11 ± 0.02). Supercritical flow is typical for situations where water swiftly passes over boulders in thin layers, which exactly fits the preferred microhabitats of *Drusus biguttatus* and its favored food source, epilithic algae, and biofilm. In contrast, near-critical and critical flow (*Fr* ~ 1) is most efficient for microhabitats of net-spinning and filtering benthic macroinvertebrates, such as hydropsychid caddisflies and blackfly larvae. The tops and sides of boulders in such zones of converging flow enable them to efficiently gather suspended particles (Wetmore et al., 1990). At subcritical conditions with *Fr* < 1, disturbance waves can travel upstream (Gordon et al., 1992). Peckarsky (1980) showed that mayflies can detect the presence of their stonefly predators by non-contact chemical cues; at subcritical flow, this vital information could also be transported upstream via waves propagated by the large sediment particles the predator is sitting, thereby enlarging the predator detection range. The same mechanism applies to kairomones emitted by predacious fire salamander larvae and their invertebrate prey (Oberrisser & Waringer, 2011). The drag force on a submerged or partially submerged body due to gravity surface waves also depends on *Fr*. In fact, the gravity surface waves which arise due to a submerged body in shallow water streams can cause downward lift forces on the body which increase with decreasing relative submergence depth (Dawson, 2014). Such downward lift forces from free surface waves could potentially assist in resisting drag forces.

As Vogel (1981) has pointed out, the lower the Reynolds number, the higher the relative amount of viscous drag. For a circular cylinder, pressure drag constitutes 57% of the total drag at *R* = 10 but 97% at *R* = 10,000 (Vogel, 1981). Thus, Statzner (1988) concluded that skin friction drag is the major component of total drag on young individuals but, as an individual grows, the proportion of pressure drag increases. As friction drag is also positively correlated with the amount of exposed body (or case) surface, a hemisphere with its favorable volume/surface ratio would suffer the lowest total drag at low Reynolds numbers, whereas a half-streamlined body with a lengthened and flattened leading edge, thus reducing the projected area, would be best suited to minimize pressure drag at high values of *R* (Statzner, 1988; Statzner et al., 1988). In the present study and for pooled data including all 31 series of measurements, we observed a mean Reynolds number (± 95% CL) of 3956 ± 72, indicating that pressure drag is by far predominant. However, since benthic macroinvertbrates are rather mobile and, at times, may be exposed also to laminar flow, this may also have been a driving force for evolving a compromise in shape, situated somewhere between hemispheres and streamlined bodies.

## Conclusion

Our study revealed that hydraulic stresses for final instar *Drusus biguttatus* larvae are not only caused by the mean flow, but strongly modified by the fluctuating part of the velocity field and by forces via the Reynolds stresses. In fact, turbulence intensity indicated medium turbulence in 14 and high turbulence in 17 out of 31 data series. The fluctuations observed represent mainly anisotropic macroturbulences consisting of eddies created by small-scale irregularities of the stream bed. As a consequence, *Drusus* larvae had to face strong flow accelerations on average every 2.5 s which is a much higher stress than conventional mean velocity measurements would suggest. *Drusus biguttatus* was able to withstand maximum instantaneous flow velocities up to 0.69 m s^−1^ and mean drag forces up to (1120 ± 34) 9 10^−6^ N without being dislodged from the substrate. Based on the observed mean Reynolds numbers of 3956, pressure drag was by far predominant. Our study also revealed that the stabilizing force due to the submerged weight of the larvae in their cases was amazingly low: on average, only 5% of the drag force was compensated by submerged weight, whereas the remainder had to be counterbalanced by the active efforts of the larvae to remain attached to the substrate. In addition, gravity surface waves may be helpful for resisting drag. They are created by the larvae in their typical shallow water habitats and can cause downward lift forces on the body which could potentially assist in drag compensation. This is illustrated by the fact that mean Froude numbers were well in the supercritical stage which is typical for situations where water swiftly passes over boulders in thin layers.

## Figures and Tables

**Table 1:** Mean velocities *ū* (averaged over the whole fluid layer), root mean square (RMS) values (= standard deviations from the mean, equivalent to turbulence intensity *I*), and absolute maxima and minima for the 31 data series; measuring positions refer to larvae 1–9 and the positions of the probe indicated in Fig. 1; *n* = number of instantaneous measurements for each series

Data series #	Measuring position	*n*	*ū* (m s^−1^)	RMS (m s^−1^)	Max (m s^−1^)	Min (m s^−1^)
1	1FC	35	0.23	0.01	0.25	0.21
2	2FC	30	0.22	0.01	0.25	0.20
3	3FC	76	0.50	0.01	0.53	0.47
4	4RCa	49	0.55	0.03	0.62	0.47
5*	4RCb	38	0.56	0.02	0.60	0.52
6	4RL	45	0.66	0.01	0.69	0.63
7*	4RR	33	0.48	0.02	0.53	0.43
8*	4MR	42	0.52	0.02	0.56	0.49
9*	4ML	48	0.08	0.02	0.14	0.04
10*	4FL	65	0.46	0.03	0.51	0.37
11	4FC	48	0.49	0.02	0.53	0.43
12	4FR	63	0.35	0.02	0.39	0.29
13	5FC	61	0.26	0.02	0.32	0.21
14	6RR	80	0.13	0.02	0.20	0.08
15*	6RL	64	0.11	0.03	0.19	0.04
16*	6FL	67	0.18	0.02	0.24	0.13
17*	6FC	63	0.20	0.02	0.26	0.14
18	6FR	65	0.14	0.02	0.19	0.11
19	6MR	62	0.18	0.02	0.24	0.14
20	6ML	69	0.14	0.01	0.19	0.10
21*	6RC	61	0.17	0.02	0.22	0.11
22	7FC	194	0.45	0.02	0.53	0.40
23	8FC	301	0.30	0.02	0.37	0.25
24	9FR	61	0.48	0.02	0.53	0.40
25	9FC	63	0.50	0.02	0.54	0.44
26	9FL	65	0.49	0.02	0.53	0.45
27	9MR	76	0.42	0.02	0.46	0.34
28	9MC	62	0.48	0.02	0.51	0.44
29*	9ML	62	0.47	0.02	0.52	0.42
30	9RC	67	0.35	0.02	0.39	0.27
31	9RL	62	0.38	0.02	0.41	0.32

Asterisks (*) indicate data series where Shapiro–Wilk’s W tests of normality were not significantly different (*P* > 0.05), indicating that the hypothesis that the distribution is normal was approved. For measuring position 4RC, two measurement series were made (4RCa, 4RCb)

**Table 2:** Biometric parameters of 11 final instar larvae of *Drusus biguttatus*

Parameter	Mean ± 95% CL
Head width (mm)	1.41 ± 0.03
Body length (mm)	8.56 ± 0.99
Anterior case diameter (mm)	3.16 ± 0.24
Case length (mm)	10.67 ± 1.28
Projected frontal surface area (mm^2^)	7.92 ± 1.28
Fresh weight (mg)	47.64 ± 6.33
Volume (mm^3^)	42.73 ±11.67
Submerged weight (mg)	8.46 ± 5.43
Adhesive friction (× 10^−6^ N)	57.27 ± 36.74

**Table 3:** Hydraulic stress parameters acting on 9 final instar larvae of *Drusus biguttatus* (longitudinal axis aligned with flow); showing the number of measurements at intervals of 1 s (*n*), mean flow velocity (mean *u*; ms^−1^), maximum flow velocity (max *u*; ms^−1^), the number of maxima observed per series, and the drag force (× 10^−6^ N)

Larva #	*n*	mean *u* (m s^−1^)	max *u* (m s^−1^)	# of maxima per series	drag (× 10^−6^ N)
1	35	0.23 ± 0.00	0.25	1	408 ± 62
2	30	0.22 ± 0.00	0.25	1	388 ± 74
3	76	0.50 ± 0.00	0.53	4	1961 ± 227
4	431	0.45 ± 0.02	0.69	1–3	1798 ± 87
5	61	0.26 ± 0.01	0.32	1	532 ± 186
6	531	0.16 ± 0.00	0.26	1	197 ± 7
7	194	0.45 ± 0.00	0.53	1	1619 ± 363
8	300	0.30 ± 0.00	0.37	2	720 ± 229
9	518	0.45 ± 0.01	0.54	1–7	1581 ± 33
Pooled data	2176	0.35 ± 0.01	0.69	1–7	1120 ± 34

All measurements were made at front center of the larvae except at larval positions # 4, 6, and 9 where 7–8 additional measurements were conducted, as shown in Fig. 1
